# Epidemiology of norovirus infections among diarrhea outpatients in a diarrhea surveillance system in Shanghai, China: a cross-sectional study

**DOI:** 10.1186/s12879-015-0922-z

**Published:** 2015-04-15

**Authors:** Ying Xue, Hao Pan, Jiayu Hu, Huanyu Wu, Jian Li, Wenjia Xiao, Xi Zhang, Zheng’an Yuan, Fan Wu

**Affiliations:** Department of Infectious Disease Control and Prevention, Shanghai Municipal Center for Disease Control and Prevention, No. 1380, West Zhongshan Road, Shanghai, 200336 China

**Keywords:** Human norovirus, Diarrhea, Surveillance, Epidemiology, Sporadic, All age groups, rRT-PCR

## Abstract

**Background:**

Norovirus is an important cause of gastroenteritis both in children and adults. In China, few studies have been conducted on adult populations. This study aimed to determine the contribution of norovirus to gastroenteritis, characterize the features of norovirus infections, compare them with other pathogens, and test the effectiveness of the surveillance system.

**Methods:**

A citywide surveillance network on diarrhea patients was established. Samples were collected with intervals from both children and adults among diarrhea outpatients in hospitals and tested for viruses using rRT-PCR and for bacteria in CDCs. Patient information was acquired through interviews and recorded into a dedicated online system. The Pearsonχ^2^ test, multivariate logistic regression models and discriminant models were fitted into its comparisons with the non-norovirus group and other pathogens.

**Results:**

Norovirus was detected in 22.91% of sampled diarrhea patients. The seasonal distribution of norovirus infections was different from non-norovirus patients (p < 0.001), with a half-year peak. Higher proportions of males (p = 0.001, OR = 1.303, 95% CI = 1.110-1.529), local citizens (p < 0.001) and officials/clerks (p = 0.001, OR = 1.348, 95% CI = 1.124-1.618) were affected with norovirus when compared with non-norovirus patients. Diarrhea patients affected with norovirus featured nausea (p < 0.001, OR = 1.418, 95% CI = 1.176-1.709) and vomiting (p < 0.001, OR = 1.969, 95% CI = 1.618-2.398), while fewer manifested fever (p = 0.046, OR = 0.758, 95% CI = 0.577-0.996) and abdominal pain (p = 0.018, OR = 0.815, 95% CI = 0.689-0.965). Children were more vulnerable to rotavirus (p = 0.008, OR = 1.637, 95% CI = 1.136-2.358) and bacteria (p = 0.027, OR = 1.511, 95% CI = 1.053-2.169) than norovirus. There was a seasonal difference between the GI and GII genotypes (p < 0.001). Officials or clerks were more easily affected with GI than GII (p = 0.006, OR = 1.888, 95% CI = 1.205-2.958).

**Conclusions:**

This study was based on a citywide hospital-sentinel surveillance system with multiple enteric pathogens included. Norovirus was recognized as the most prevalent enteric pathogen in Shanghai. The seasonal peak was from October to April. Males had a higher prevalence than females. Local citizens and officials/clerks were more vulnerable to norovirus than other pathogens. Compared with rotavirus and bacteria, children were less frequently affected by norovirus. Nausea and vomiting were typical of norovirus, whereas fever and abdominal pain were uncommon symptoms of this pathogen. GI and GII infections were centered in different seasons. Officials and clerks were more easily affected by GI than GII.

**Electronic supplementary material:**

The online version of this article (doi:10.1186/s12879-015-0922-z) contains supplementary material, which is available to authorized users.

## Background

Diarrheal disease morbidity and mortality have been in decline globally, but around 1.7-5 billion cases of diarrhea [1] and nearly 1.7 million diarrheal deaths still occur each year [2], the great majority of which are among young children in developing countries [3].

Norovirus is a leading cause of non-bacterial gastroenteritis in both developed and developing countries [4] and is increasingly appreciated as an important cause of gastroenteritis. Norovirus is also considered to be the second most frequent cause of severe childhood gastroenteritis after rotavirus [5]. Its prevalence in children with acute gastroenteritis is in the range of 6–48% [6]. The development of molecular techniques in diagnosing has brought its epidemiological impact into sight [7]. It was concluded that an average of 570–800 deaths, 56,000-71,000 hospitalizations, 1.7-1.9 million outpatients visits, and 19–21 million total illnesses occurin the United States each year as a result of norovirus infections [8]. Although previous studies indicated that the disease was mild and self-limiting, recent studies have revealed its ability to cause more severe complications than previously expected [9,10]. In addition to human losses, the economic costs caused by norovirus infections were considerable. It is estimated that the economic burden of norovirus infections approached or exceeded US$284 million annually in health care charges in the United States [11].

The increasing number of global public health concerns caused by norovirus in recent years has largely been driven by an abundance of reported outbreaks [8]. A systematic literature review identified >900 published reports of laboratory-confirmed norovirus outbreaks during 1993 ~ 2011 [12]. However, the predominance of outbreak reports was mainly due to deficient sporadic data, because norovirus is not routinely tested in clinical settings due to high molecular method requirements. Because of this, the characterization of norovirus epidemiology has been primarily performed through the analysis of outbreak data [13].

In China, acute nonbacterial gastroenteritis is also considered a severe public health problem [9]. However, very few studies have been focused on adult populations so as to illustrate the relative importance of norovirus and other enteric pathogens. In some developed countries, the typical age pattern of diarrhea mortality is reversed; diarrhea-associated deaths are 5 times more common in elderly individuals than in children [14].

Owing to the overrepresentation of studies merely in children and a lack of sporadic data on norovirus infections [9,13,15], the role of norovirus as the etiological agent in acute diarrhea needs to be further defined. The objectives of this study were to determine the infection rate of norovirus among diarrhea patients in Shanghai and describe the epidemiological characteristics of norovirus infections in an attempt to test the effectiveness of the surveillance system and make progress towards its future popularization.

## Methods

### Background information

Shanghai is a metropolis with a population of more than 23 million as of 2010. Of the total population, 62.61% were locals [16] and the sex ratio (male: female) of the city was 1.06:1. The population of the elderly (>60y) was 3.47 million (15.07%) and for the elderly, the sex ratio (male: female) was 0.92:1. The average life expectancy in 2010 was 82.13 years old [17]. There are 17 administrative districts in Shanghai. All of the hospitals in the surveillance system have enteric disease clinics for diarrhea patients for quarantine purposes.

### Surveillance system

The surveillance first began with 6 adult hospital sentinels in May 2012, with a child sentinel (specialized city hospital) joining in October 2012, and 16 additional adult hospital sentinels in August 2013.

#### General framework

The surveillance system consisted of three levels: hospital sentinels for case finding, sampling and information collection; district-level centers for disease control and prevention (CDCs) for sample testing; and the municipal CDC for management and quality control. The three levels could share information through a dedicated online system.

#### Case definition

Surveillance subjects were defined as those who visited the enteric disease clinics of sentinel hospitals, with 3 or more loose or liquid stools per day [the definition of diarrhea by the World Health Organization (WHO) [18]. Norovirus-affected patients were defined as those whose stool samples were norovirus-positive, including patients with sole-infections and co-infections.

#### Sampling

To date, a total of 23 hospital sentinels were sampled using Probability Proportionate to Size (PPS) Sampling across all hospital types and spread over all 17 districts in Shanghai. The total sample size was calculated on the basis of the number of diarrhea patients of sampled hospitals in Shanghai and previous local studies on enteric pathogens. Systematic sampling was used for sample collection. Different intervals were allocated to different sentinel hospitals under a comprehensive calculation of the hospital’s location, classification and annual number of diarrhea patients.

#### Information collection

All surveillance subjects were interviewed by doctors. General, epidemiological and medical information was obtained and recorded into the online system. Outbreak sources were excluded as much as possible via inquiry.

#### Laboratory tests

Stool samples were collected from surveillance subjects in designated intervals by trained medical staff. Approximately 8 ~ 10 g (mL) of stool was collected and then dispensed into two containers: a tube with Cary-Blair (C-B) culture medium for bacteria testing and a sterile box for virus testing. Nucleic Acid was extracted from fecal specimens (20% wt/vol or vol/vol suspensions) using the QIAamp Viral RNA Kit (Qiagen, Hilden, Germany). Norovirus detection was performed using a real-time Reverse Transcription -Polymerase Chain Reaction (rRT-PCR) method. The viral RNA was reverse transcribed using M-MLV (Promega, Madison, WI) according to the manufacturer’s instructions. The primers (Cog1F/Cog1R) and the probes (Ring1A/Ring1B) were used to detect norovirus GI, and the primers (Cong2F/Cog2R) and probe (Ring2) were used to detect norovirus GII [19]. Probes Ring1A/Ring1B and Ring2 were each labeled with FAM and HEX at 50 extremities. The final reaction volume was 20 μl, consisting of 1 μl RNA and 19 μl RT-PCR master mix. The concentrations of the primers and probes were as follows: for the GI assay, 0.2 μM probe and 0.4 μM each primer; for the GII assay, 0.4 μM probe and 0.4 μM each primer. The thermal cycling conditions: RT for 30 min at 55°C, followed by denaturation at 95°C for 30s, amplification for 45 cycles, followed by denaturation at 95°C for 10s, and annealing-extension at 60°C for 60s. A negative control containing diethyl pyrocarbonate (DEPC) water and two positive controls containing the RNA of norovirus GI and GII were included in each PCR run. Samples were scored as positive if cycle threshold values were less than 40 and positive and negative controls showed the expected values.

Apart from norovirus detection, all of the samples were also screened for other viruses (astrovirus, sapovirus, rotavirus and enteric adenovirus), and for bacteria [Vibrio cholerae, Shigella, Salmonella, Vibrio parahemolyticus, Campylobacter jejuni (C. jejuni), Yersinia enterocolitica, Campylobacter coli (C. coli), Enteropathogenic escherichia coli (EPEC), Enterotoxigenic escherichia coli (ETEC), Enterohemorrhagic escherichia coli (EHEC), Enteroaggregative escherichia coli (EAggEC), Enteroinvasive escherichia Coli (EIEC)]. Astrovirus, sapovirus and rotavirus were detected using rRT-PCR and enteric adenovirus was detected using real-time PCR, all of which was performed using the appropriate respective commercial kits (Shanghai Zhijiang Biotechonology Co., Ltd.) according to the instructions provided by the manufacturer. Bacteria were isolated using different mediums at proper temperatures after preparation. The mediums included ChromID Vibrio and TCBS for Vibrio cholera and Vibrio parahemolyticus, MAC for Escherichia coli, XLD for Shigella and Salmonella, etc.. Bacteria were identified using biochemical tests. An automatic biochemical identification system was used for Escherichia coli. Serum agglutination tests were employed to subtype Shigella, Salmonella, Vibrio cholera and Escherichia coli.

Samples were taken as a part of standard medical care. All laboratory results were recorded and viewed using the online system.

### Ethics

The study protocol was reviewed and approved by the Human Research Ethics Committee of the Shanghai Municipal Center for Disease Control and Prevention.

### Statistical analysis

Data analyzed in the study were from May 1, 2012 to April 30, 2014 (date of visit) and downloaded on May 26, 2014. The division of age groups conforms to the *Convention on the Rights of the Child* and WHO standards. The definition of seasons was determined by the climatic characteristics of Shanghai. Differences in discrete variable levels were examined using the Pearsonχ^2^ test. Fisher’s test was used when the expected value was less than 5 or when the p value was close to the level of the test. A multivariate logistic regression model was used to seek characteristic differences as integrated in a clinical setting (NoV+ vs NoV-; NoV+ vs RV+; NoV+ vs bacteria+; genotype GI vs GII). Discriminant analysis was used to identify the symptom complex of norovirus infections. Two-tailed P < 0.05 was considered statistically significant. Version 17.0 of the SPSS software package was used for all analyses (SPSS, Inc., Chicago, IL).

## Results

### General characterization

During the 2-year study period, a total of 44595 diarrhea patients were studied. The mean (±SD) age of the study subjects was 43.51 (±19.06 ) years and 21657 (48.56%) were male. Among the surveillance subjects, a total of 3941 samples (8.84%) were detected (duplicated samples excluded). There were 2114 positive samples detected (positive rate 53.64%) and 903 (detection rate 22.91%) patients were positive for norovirus (referred to as “NoV + ”), consisting of GI (94, 10.41%), GII (769, 85.16%) and co-infections of GI and GII (40, 4.43%). Co-infections of norovirus and other viruses or bacteria were confirmed in 91 cases (2.31%). Excluding co-infection samples, 2947 samples (74.78%) were confirmed as negative for all tested pathogens or positive for other enteric pathogens (referred to as “NoV -”). The positive rates of other enteric pathogens were as follows (excluding co-infections): Shigella 0.51%, Salmonella 3.63%, Vibrio parahemolyticus 3.93%, C. jejuni 0.66%, Yersinia enterocolitica 0.05%, C. coli 0.08%, EPEC 0.74%, ETEC 0.86%, EAggEC 0.10%, EIEC 0.03%, astrovirus 2.54%, rotavirus 10.05%, sapovirus 2.36%, and enteric adenovirus 0.53%.

### NoV(+) sample features and comparison with NoV(−)

#### Epidemiological analysis

**Figure 1:**
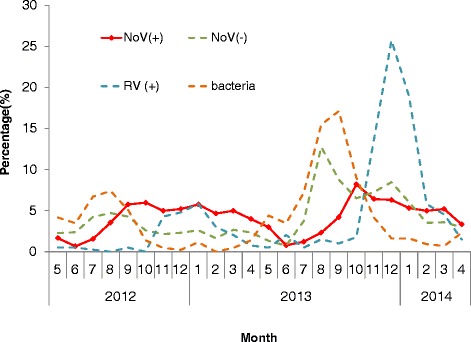
Monthly infection percentage of NoV(+), NoV(−), RV(+) and bacteria. The percentage of the infections detected in this particular month out of the whole study period was calculated.

**Figure 2:**
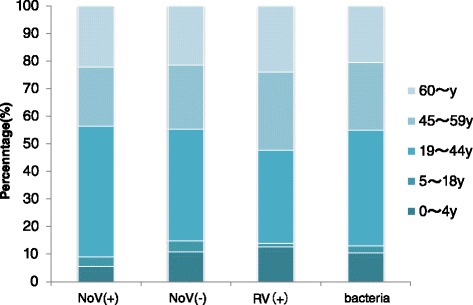
Age distribution of NoV(+), NoV(−), RV(+) and bacteria patients. From May 2012 to September 2012, only 6 sentinels were under surveillance. A child sentinel joined in October 2012. In August 2013, the number of sentinels expanded to 23.

Norovirus was detected throughout the year, and the prevailing season lasted as long as half a year (from October to April) (See Figure 1). The seasonal distribution of NoV(+) detection was different from NoV(−) (p < 0.001), but no difference was found between autumn (September-November) and winter (December-February) (p = 0.117). Norovirus spanned all ages, from 0 to 94 years old. The proportion of the child population of NoV(+) patients seemed smaller than that of NoV(−) ones (See Figure 2), but the difference was not statistically significant in a logistic regression model. The sex ratio (male: female) was 1.18:1, with a higher male proportion in the NoV(+) group (p = 0.001, OR = 1.303, 95% CI = 1.110-1.529). The proportion of local citizens infected with norovirus was higher than that of non-norovirus patients (p < 0.001). Norovirus had a higher chance of appearing in: officials/clerks (p = 0.001, OR = 1.348, 95% CI = 1.124-1.618) and a lower chance of appearing in farmers/migrant laborers (p = 0.007, OR = 0.243, 95% CI = 0.087-0.680).

#### Stratification analysis

**Figure 3:**
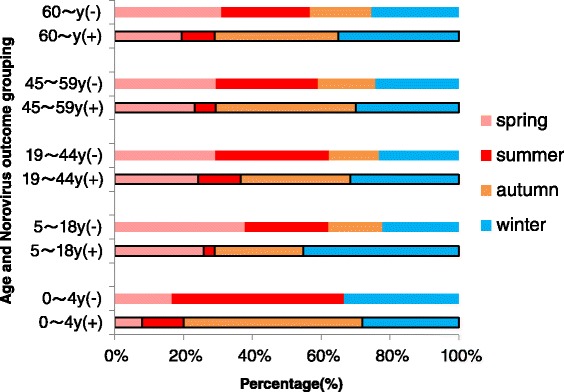
Seasonal distribution of NoV(+) and NoV(−) patients within age groups.

In an age stratification analysis, it was discovered that NoV(+) and NoV(−) patients had statistically different seasonal distributions for each age group (0 ~ 4y, p = 0.017; 5 ~ 18y, p = 0.005; 19 ~ 44y, 45 ~ 59y, >60y, all p < 0.001) (See Figure 3), and a significant seasonal difference among NoV(+) patients of different age groups could also be determined (p ≈ 0.027). While a difference in the proportions of male and female NoV(+) patients could be found among different age groups (p = 0.016): in the children and youth groups (<44y), males were dominant, and in the middle-aged and elderly groups (>45y), vice versa (p < 0.001, OR = 1.586, 95% CI = 1.216-2.067), the gender distribution did not differ much from NoV(−) patients, except in the youth (19-44y) group, where male patients had a higher proportion of infections (p = 0.024, OR = 1.302, 95% CI = 1.041-1.629).

#### Analysis of exposure history

When compared with NoV(−), a higher proportion of NoV(+) patients had a history of consuming suspicious food within 5 days before onset (p = 0.001, OR = 1.319, 95% CI = 1.124-1.550), while a lower proportion had an enteric disease history 6 months prior (p = 0.048, OR = 0.341, 95% CI = 0.117-0.992).Although a large percentage (53.09%) of the children (<18y) group kept or had contact with pets, in a univariate χ^2^ test, there was no statistically significant difference between NoV(+) and NoV(−) patients (p = 0.451) within this group.

#### Clinical feature analysis

NoV(+) diarrhea patients featured nausea (p < 0.001, OR = 1.418, 95% CI = 1.176-1.709) and vomiting (p < 0.001, OR = 1.969, 95% CI = 1.618-2.398), while fewer reported fever (p = 0.046, OR = 0.758, 95% CI = 0.577-0.996) and abdominal pain (p = 0.018, OR = 0.815, 95% CI = 0.689-0.965) when compared with NoV(−) patients. In a discriminant analysis, the relationship between symptoms and norovirus infections was also studied. The combination of nausea and vomiting (especially lasting over three days) was typical of norovirus infections, while fever (especially high fever) and abdominal pain were adverse determining factors (p < 0.001). General, epidemiological and clinical comparisons are listed (see Additional file 1).

### Comparisons with rotavirus and bacterial infections

A total of 396 samples were confirmed to have rotavirus infections (10.05%), and 432 samples had bacterial infections (10.96%) (co-infections excluded). Comparisons with norovirus regarding their general, epidemiological and clinical features with norovirus are listed (see Additional files 2 and 3).

The seasonal difference between norovirus and rotavirus detection was obvious (p < 0.001): rotavirus mainly occurred in the coldest seasons (from November to February) (See Figure 1). There was also a difference in age distribution between the two viruses (p = 0.002): rotavirus affected children more (p = 0.008, OR = 1.637, 95% CI = 1.136-2.358) (See Figure 2). The proportion of males (p = 0.004, OR = 1.475, 95% CI = 1.133-1.919) and local citizens (p < 0.001) with confirmed norovirus was higher than those with rotavirus. Norovirus and rotavirus were detected from hospitals of different types (p = 0.006). Rotavirus-affected patients had a higher proportion of suspicious food history (p < 0.001, OR = 2.006, 95% = 1.447-2.781). Patients affected with norovirus were more likely to manifest vomiting (p < 0.001, OR = 1.860, 95% CI = 1.373-2.520), but less likely to manifest fever (p = 0.034, OR = 0.626, 95% CI = 0.406-0.964).

The seasonal difference between norovirus and bacteria was more obvious (p < 0.001): bacteria were mostly found in warm seasons (from July to September) (See Figure 1). Age was also an influencing factor p = 0.037): a higher proportion of children were infected with bacteria than norovirus (p = 0.027, OR = 1.511, 95% CI = 1.053-2.169) (See Figure 2). Local citizens had a higher proportion of norovirus infections (p = 0.003). For the following occupations, there was a lower prevalence of norovirus than of bacteria: kindergarten/home-stay children (p = 0.033, OR = 0.090, 95% CI = 0.010-0.822) and farmers/migrant laborers (p = 0.008, OR = 0.180, 95% CI = 0.051-0.643). Norovirus-affected patients had a higher proportion of suspicious food history (p < 0.001, OR = 1.686, 95% CI = 1.266-2.244). Compared with bacteria, norovirus patients less frequently manifested fever (p < 0.001, OR = 0.428, 95% CI = 0.288-0.635) and abdominal pain (p < 0.001, OR = 0.405, 95% CI = 0.299-0.549), but more frequently manifested nausea (p = 0.001, OR = 1.735, 95% CI = 1.247-2.412) and vomiting (p = 0.006, OR = 1.620, 95% CI = 1.149-2.286).

### Features of GI and GII genotypes

769 GII strains were detected concomitant with 94 GI ones. The Pearsonχ^2^ test indicated that the seasonal distribution of two genotypes was different (p < 0.001), with GI highest in spring (March to May) (44.68%) and GII highest in autumn (September to November) (40.44%). Those who work as officials or clerks had a higher possibility of being affected by GI (42.55%) than GII (25.75%) (p = 0.001, OR = 2.136, 95% CI = 1.368-3.289). GI-affected patients seemed to have eaten in a restaurant more often (3.19%) than GII-affected patients (0.65%) (p = 0.047, OR = 5.037, 95% CI = 1.185-21.277). The rate of patients who had consumed contaminated seafood within five days before onset was higher in GI (17.02%) than in GII (10.92%) patients (p = 0.019, OR = 2.294, 95% CI = 1.164-4.525). There were also slight differences in the clinical features of the two genotypes: nausea (54.26% of GI and 42.65% of GII, p = 0.037, OR = 1.595, 95% CI = 1.031-2.439), diarrhea lasting less than three days (87.23% of GI and 81.14% of GII, p = 0.035, OR = 4.008, 95% CI = 0.959-16.667), and hyperactive bowel sounds (37.23% of GI and 23.28% of GII, p = 0.003, OR = 1.955, 95% CI = 1.250-3.077).

In a logistic regression model, officials or clerks were more easily affected with GI than GII (p = 0.006, OR = 1.888, 95% CI = 1.205-2.958). Seasonal differences were statistically significant in both genotypes (p < 0.001). A higher proportion of patients who had eaten in a restaurant was affected with GI than GII (p = 0.048, OR = 4.717, 95% CI = 1.013-21.960).

### Co-infection samples

91 norovirus co-infection samples were discovered (excluding co-infections of norovirus GI and GII): 21 with rotavirus, 18 with astrovirus, 15 with sapovirus, 10 with Salmonella, 7 with EPEC, 5 with Vibrio parahemolyticus, 5 with adenovirus, 3 with C. jejuni,2 with C. coli, 1 with Shigella, 1 with EAggEC and 3 triple co-infections.

## Discussion

Acute gastroenteritis is one of the most common diseases reported in humans. Norovirus is not only the leading cause of non-bacterial gastroenteritis outbreaks, but it is also currently recognized as a major cause of sporadic gastroenteritis in both children and adults [7]. In China, acute nonbacterial gastroenteritis is also considered to be a severe public health problem. However, most studies have mainly focused on norovirus infections of children, while little research has been conducted in adult populations to clarify the importance of norovirus [9]. In addition, materials and analyses with regards to norovirus have mostly been obtained from outbreak resources [13].

This study was the first in Shanghai to be concerned with sporadic norovirus infections of the whole population. It was based on a diarrhea disease surveillance system in Shanghai, which began in 2012. Compared with other studies[9,15], this study distributed sentinels across the city and used systematic sampling, which are better able to better represent and be extrapolated to the city’s population by avoiding the influence of clusters and season-specific cases; the incidence rate and disease burdens could be calculated in future studies. The positive rate of norovirus was 22.91% among 3941 diarrhea patients, which was quite close to the result of a previous study in Beijing (26.4% among acute non-bacterial gastroenteritis patients) [9] and another in Shenzhen (21.4% in acute gastroenteritis patients) [20]. In this study, the norovirus infection rate was the highest among all pathogenswhen co-infections were excluded.

Previous studies have reported that norovirus mainly peaked in winter or cold seasons [21,22]. In this study, the result verified this conclusion, as more strains were detected from October to April (when the weather was comparatively cold in Shanghai). Interestingly, an autumn peak was as distinct as the winter one, which was in concert with another study in Beijing [23] (despite the fact that autumn in Beijing is colder than Shanghai). This could perhaps be explained by the immunity barrier to the current epidemic strain set up by the population during the epidemic season in autumn. The relationship of norovirus infections and temperature should be further explored in the future studies. For different age groups, the seasons when people were vulnerable to norovirus seemed different in a univariateχ^2^ test, but not enough samples could be included in a logistic regression model. More studies could be made if enough data were acquired.

Other than previous studies, which concluded that young children and elderly people were more vulnerable than other age groups [10,24], our study discovered that the highest detection rate was found in the youth group (19-44y) (25.97%), and the lowest in the children group (13.30%), with the elderly group in the middle (23.50%), whereas in a logistic model, the age distribution of NoV(+) patients could not be proved to be different from NoV(−) patients. Age distribution differences were found to be significant in NoV(+) vs RV(+) and NoV(+) vs bacteria(+) comparisons, which are stated below.

Males were found to more often be affected by norovirus when compared with other enteric pathogens. Although 0 ~ 44y males accounted for a higher proportion in the NoV(+) group and >45y a lower proportion in an age stratification analysis, the distribution seemed to be a general characteristic of all diarrhea patients.

It was observed that local citizens and officials/clerks had a higher proportion of norovirus infections, while immigrants and farmers/migrant laborers a lower proportion. There originally existed associations between the residency and occupation results, and yet it still seemed that norovirus was a more “urban” virus.

A history of consuming suspicious food within 5 days before onset was more commonly recorded among norovirus affected patients than non-norovirus ones. Nevertheless, the fact that a large part of non-norovirus diarrhea patients might have had physiological diarrhea or non-communicable enteric diseases might influence the outcome. It was also found that rotavirus-affected patients had a higher proportion of suspicious food history than norovirus patients, while bacteria had a lower proportion. Unfortunately, though specific food category information was gathered, the valid sample size was not large enough to be included in a logistic regression model. Further research regarding specific food risk factors could be made in future studies.

Some studies recognized diarrhea, vomiting and fever as the most common symptoms of norovirus-affected patients [13,24-26]. Although in this study it was proved that norovirus was distinguished by diarrhea (automatically included), nausea, and vomiting among diarrhea patients, fever was less commonly seen in NoV(+) patients when compared with NoV(−) ones. Other studies also claimed a lower occurrence of fever in norovirus patients than in rotavirus ones [21,22,27], but their rate was still much higher than what was reported in this study (only 8.86% norovirus-affected patients experienced fever). This was probably because febrile patients tend to visit fever clinics in Chinese hospital settings. Abdominal pain was also identified as a rare symptom of norovirus, which was similar to the result of a previous study [24]. The clinical feature results produced in a logistic model were analogous to those in a discriminant analysis.

Comparisons between NoV(+) vs RV(+) and NoV(+) vs bacteria(+) were also made in this study to help enhance the cognition of the disease and provide evidence for a rough diagnosis. The results were broadly in line with the NoV(+) vs NoV(−) comparison, but some new conclusions were also drawn: rotavirus occurred in an even colder climate, and bacteria mainly appeared in hot seasons. Age distribution differences were significant here: children were more vulnerable to rotavirus and bacteria than norovirus. Although the difference in the proportion of patients showing abdominal distention in norovirus and bacterial infections was not significant (p = 0.053), observations should be continued when more data is obtained.

Norovirus GII is predominantly responsible for acute diarrhea worldwide, as described in most studies [10,24], and our findings (10.41% GI, 85.16% GII, 4.43% mix of both genotypes) were consistent with them. Our research also did further studies on the comparison of features between two genotypes: GI prevailed in spring while GII in autumn. This could be caused by variant alternation with seasonal changes. It was found in a univariate analysis that a higher proportion of GI-affected patients had the symptoms of nausea, diarrhea lasing for less than three days and hyperactive bowel sounds, whereas the results were not supported by the multivariate model. The influence of consuming seafood within five days before onset was also not backed up by the multivariate model. On the other hand, the multivariate model supported the univariate conclusions that the occupations of officials/clerks were a risk factor for infection with GI variants other than GII, though the power of the logistic model might be slightly compromised because of the small sample size of GI cases. Having eaten in a restaurant could not be regarded as a risk factor here as the confidence interval was too wide. In order for results to be revealed by either univariate or multivariate models, there needs to be more data for tests in the future.

The limitations of this study should be considered. First, data were gathered through 23 hospitals and 17 laboratories. Though testing methods and materials were unified, there was still a chance of bias caused by the different levels and conditions of laboratories (as suggested above). Admission rate bias should also be taken into account as patients visiting hospitals of different levels or in different regions were quite different. Furthermore, variations in the sentinel numbers would certainly affect the observation of seasoning, though it could be alleviated by making a comparison with non-norovirus patients. Second, only one child sentinel was enrolled and the data regarding children were quite limited. The testing power of age distribution might therefore be undermined. Next year we will enlarge the range of surveillance in children and include more data. Third, RNA sequencing of the positive samples was not done in this study. Since different features could possibly be found between GI and GII genotypes, this issue is deserving of further research with regard to particular strains and variants. Fourth, the recall bias of epidemiological information was difficult to avoid. The information on exposure history was primitive in this study. For example, water contamination was an important cause of norovirus outbreaks [27-31], and in another study, drinking spring water was reported as a risk factor [24]. However, in this study, only 7 out of 3941 diarrhea patients reported drinking contaminated water and none of them were affected with norovirus. Meanwhile, very few data from general laboratory examination results were recorded. Perhaps if more meaningful variables of this sort of information were studied in the model, the power of the test would be greater. Fifth, because of the small sample size and short surveillance time, we did not perform further research among sole-infections and co-infections and other stratification analyses.

## Conclusions

This was the first study on norovirus among all age groups in Shanghai. In this study, several meaningful conclusions were acquired: Norovirus was the most frequent enteric pathogen in Shanghai during the past two years; the epidemic season of norovirus was October ~ April in Shanghai; the norovirus infection proportion in children (<18y) was found to be lower than rotavirus and bacteria; Males had a higher proportion of norovirus infections than females; higher proportions of local citizens and officials/clerks were infected with norovirus than other pathogens; Norovirus could be characterized by nausea and vomiting among diarrhea patients, but fever and abdominal pain were rare symptoms; the GI genotype prevailed in spring while GII in autumn; It was easier for officials/clerks to be affected with GI than GII. These results might serve to promote diagnosis in a clinical setting, especially for medical staff to detect outbreaks and trace sources early. For public health workers, the results could help determine focus timing and populations of norovirus infections.

The study was based on a diarrhea surveillance system combining case finding in hospitals and laboratory testing in CDCs into one platform via a dedicated online system. The results of the norovirus survey proved that the surveillance system was running well. Other credible and constructive results on general, epidemiological and clinical features were generated. In the future, more improvements on epidemiological data, medical recording and RNA sequencing should be made based on the system. Nevertheless, the system still has reference value for other regions.

## Additional files

Additional file 1: Table S1. Epidemiology and clinical features by examining NoV(+) and NoV(−) patients. Additional file 2: Table S2. Epidemiology and clinical features by examining NoV(+) and RV(+) patients. Additional file 3: Table S3. Epidemiology and clinical features by examining NoV(+) and bacteria(+) patients.

## Abbreviations

rRT-PCR: Real-time Reverse Transcription -Polymerase Chain Reaction
CDC: Center for Disease Control and Prevention
OR: Odds Ratio
CI: Confidence Interval
WHO: World Health Organization
PPS: Probability Proportionate to Size
C-B: Cary-Blair
DEPC: Diethyl pyrocarbonate
C. jejuni: Campylobacter jejuni
C. coli: Campylobacter coli
EPEC: Enteropathogenic escherichia coli
ETEC: Enterotoxigenic escherichia coli
EHEC: Enterohemorrhagic escherichia coli
EAggEC: Enteroaggregative escherichia coli
EIEC: Enteroinvasive escherichia coli
NoV+: NoV(+): norovirus positive
NoV-: NoV(−), Norovirus negative
RV+: RV(+), Rotavirus positive
