# Supplementary material for: Epidemiology of norovirus infections among diarrhea outpatients in a diarrhea surveillance system in Shanghai, China: a cross-sectional study
Source: BMC Infect Dis. 2015 Apr 15;15:183. doi: 10.1186/s12879-015-0922-z (PMC4438334; doi:10.1186/s12879-015-0922-z)
Supplement: Additional file 1: Table S1. — Epidemiology and clinical features by examining NoV(+) and NoV(−) patients. [file 12879_2015_922_MOESM1_ESM.pdf]

Table 1. Epidemiology and clinical features by examining NoV(+) and NoV(-) patients

Parameter	NoV(+) n=903	NoV(-) n=2947	P^a	OR^a	95%CI^a	P^b	OR^b	95%CI^b
**Detection Rate (%)**	22.91	74.78						
**Season**								
Spring(Mar.~May)	200(22.15)	448(15.20)						
Summer (Jun.~Aug.)	91(10.08)	842(28.57)	<0.001	-	-	<0.001	-	-
Autumn (Sep.~Nov.)	321(35.55)	931 (31.59)						
Winter (Dec.~Feb.)	291(32.23)	726(24.64)						
**Age**								
0~4y	50(5.54)	318(10.79)						
5~18y	31(3.43)	120(4.07)						
19~44y	428(47.40)	1192(40.45)	<0.001	-	-	0.222	-	-
45~59y	194(21.48)	683(23.18)						
60~y	200(22.15)	634(21.51)						
**Gender**								
Male	489(54.15)	1489(50.53)	*0.057*	*1.157*	0.996-1.343	0.001	1.303	1.110-1.529
Female	414(45.85)	1458(49.47)						
**Residency**								
Local	805(89.15)	2449(83.10)	<0.001	1.670	1.326-2.104	<0.001	-	-
Immigrant	98(10.85)	498(16.90)						
**Occupation**[d]								
Officials/clerks	253(28.02)	548(18.60)				0.001	1.348	1.124-1.618
Kindergarten /home-stay children	47(5.20)	310(10.52)	<0.001[c]	-	-	0.284	-	-
Farmers/migrant laborers	4(0.44)	62(2.10)				0.007	0.243	0.087-

								0.680
Missing data	599(66.33)	2027(68.78)						
**Hospital Type Classification**								
Community health center	113(12.51)	448(15.20)						
District hospital	345(38.21)	1157(39.26)	*0.057*	-	-	0.339	-	-
General hospital of the city	445(49.28)	1342(45.54)						
**Suspicious food(in 5 days before onset)**								
Yes	426(47.18)	1112(37.73)	<0.001	1.474	1.268-1.713	0.001	1.319	1.124-1.550
No	477(52.82)	1835(62.27)						
**Raising or contact with pets**								
Yes	210(23.26)	663(22.50)	0.634	1.044	0.875-1.246	-	-	-
No	693(76.74)	2284(77.50)						
**Travel history**								
Yes	15(1.66)	31(1.05)	0.140	1.589	0.854-2.957	-	-	-
No	888(98.34)	2916(98.95)						
**Restaurant dining**								
Yes	8(0.89)	37(1.26)	0.366	0.703	0.326-1.515	-	-	-
No	895(99.11)	2910(98.74)						
**Similar patients nearby**								
Yes	3(0.33)	11(0.37)	1.000	0.890	0.248-3.196	-	-	-
No	900(99.67)	2936(99.63)						
**Suspicious water**								
Yes	0(0.00)	6(0.20)	0.347	-	-	-	-	-

	No	903(100.00)	2941(99.80)						
**Antibiotics take-in**									
	Yes	52(5.76)	165(5.60)	0.856	1.030	0.747-1.420	0.631	-	-
	No	851(94.24)	2782(94.40)						
**Enteric disease history(in 6 months before)**									
	Yes	4(0.44)	36(1.22)	*0.058*	*0.360*	*0.128-1.014*	0.048	0.341	0.117-0.992
	No	899(99.56)	2911(98.78)						
**Fever**									
	Yes	80(8.86)	308(10.45)	0.164	0.833	0.643-1.078			
	No	823(91.14)	2639(89.55)				0.046	0.758	0.577-0.996
	37.5℃≤t≤39.0℃	78(97.50)	294(95.45)	0.541	1.857	0.413-8.344	-	-	-
	t>39.0℃	2(2.50)	14(4.55)						
**Nausea**									
	Yes	404(44.74)	891(30.23)	<0.001	1.868	1.603-2.177	<0.001	1.418	1.176-1.709
	No	499(55.26)	2056(69.77)						
**Dehydration**									
	No	883(97.79)	2864(97.18)						
	Mild	20(2.21)	79(2.68)				0.339	-	-
	Moderate	0(0.00)	3(0.10)						
	Severe	0(0.00)	1(0.03)						
**Vomiting**									
	Yes	303(33.55)	515(17.48)	<0.001	2.385	2.017-	<0.001	1.969	1.618-

No	600(66.45)	2432(82.52)			2.820			2.398
1~2days	268(88.45)	413(80.19)	0.320	1.687	0.595-4.787	-	-	-
⩾3days	5(1.65)	13(2.52)						
Missing data	30(9.90)	89(17.28)						
<3 times /day	176(58.09)	334(64.85)	*0.051*	*0.745*	*0.556-0.997*	-	-	-
≥3 times /day	126(41.58)	178(34.56)						
Missing data	1(0.33)	3(0.58)						
**Abdominal Pain**								
Yes	417(46.18)	1370(46.49)	0.871	0.988	0.851-1.147	0.018	0.815	0.689-0.965
No	486(53.82)	1577(53.51)						
Persistent	22(5.28)	64(4.67)	0.614	1.136	0.691-1.869	-	-	-
Paroxymal	395(94.72)	1306(95.33)						
**Abdominal Distention**								
Yes	140(15.50)	352(11.94)	0.003	1.353	1.094-1.672	0.158	-	-
No	763(84.50)	2595(88.06)						
**Diarrhea**								
<3 times /day	70(7.75)	188(6.38)	0.168	1.222	0.918-1.626	-	-	-
≥3 times /day	819(90.70)	2688(91.21)						
Missing data	14(1.55)	71(2.41)						
1~2 days	741(82.06)	2113(71.70)	0.001	2.247	1.704-2.962	-	-	-
≥3 days	64(7.09)	410(13.91)						
Missing data	98(10.85)	424(14.39)						
**Stool Appearance**								
Watery	700(77.52)	2019(68.51)						
Loose	172(19.05)	671(22.77)	<0.001	-	-	-	-	-
Mucous	14(1.55)	138(4.68)						

	Bloody	1(0.11)	34(1.15)						
	Other	2(0.22)	14(0.48)						
	Missing data	14(1.55)	71(2.41)						
**Tenesmus**									
	Yes	9(1.00)	55(1.87)	0.075	0.529	0.261-1.075	0.116	-	-
	No	894(99.00)	2892(98.13)						
**Hyperactive bowel sound**									
	Yes	220(24.36)	623(21.14)	0.043	1.202	1.008-1.433	0.524	-	-
	No	683(75.64)	2324(78.86)						

Notes.

Calculation of OR: NoV(+) to NoV(-)/ the first row to the second row.

Meaningful results were underlined. The P values which were close to the level of test (p<0.075) were in italics.

Cutoff=0.235.

To analyze more comprehensive clinical features, the data of less frequent stools(<3 times/day) were also included.

- Means "not done in the analysis".

[a] Outcome by the Pearson χ^2 test or the Fisher's test.

[b] Outcome by a multivariate logistic regression model.

[c] Compared among 18 groups in the "occupation" category.

[d] Only three of 18 kinds of occupations were included and analyzed in a logistic model(as a binary variable); others were interpreted as "missing data".
