# Supplementary material for: Epidemiology of norovirus infections among diarrhea outpatients in a diarrhea surveillance system in Shanghai, China: a cross-sectional study
Source: BMC Infect Dis. 2015 Apr 15;15:183. doi: 10.1186/s12879-015-0922-z (PMC4438334; doi:10.1186/s12879-015-0922-z)
Supplement: Additional file 2: Table S2. — Epidemiology and clinical features by examining NoV(+) and RV(+) patients. [file 12879_2015_922_MOESM2_ESM.pdf]

Table 2. Epidemiology and clinical features by examining NoV(+) and RV(+) patients

Parameter	NoV(+) n=903	RV（+） n=396	P[a]	OR[a]	95%CI[a]	P[b]	OR[b]	95%CI[b]
**Detection Rate (%)**	22.91	10.05						
**Season**								
Spring(Mar.~May)	200(22.15)	39(9.85)						
Summer(Jun.~Aug.)	91(10.08)	19(4.80)	<0.001	-	-	<0.001	-	-
Autumn(Sep.~Nov.)	321(35.55)	84(21.21)						
Winter(Dec.~Feb.)	291(32.23)	254(64.14)						
**Age**								
0~4y	50(5.54)	50(12.63)						
5~18y	31(3.43)	5(1.26)						
19~44y	428(47.4)	134(33.84)	<0.001	-	-	0.002	-	-
45~59y	194(21.48)	112(28.28)						
60~y	200(22.15)	95(23.99)						
**Gender**								
Male	489(54.15)	190(47.98)	0.046	1.281	1.011-1.623	0.004	1.475	1.133-1.919
Female	414(45.85)	206(52.02)						
**Residency**								
Local	805(89.15)	333(84.09)	0.013	1.554	1.105-2.186	<0.001	-	-
Immigrant	98(10.85)	63(15.91)						
**Occupation[d]**								
Officials/clerks	253(28.02)	71(17.93)				*0.058*	-	-
Kindergarten /home-stay children	47(5.20)	46(11.62)				0.703	-	-
Farmers/migrant laborers	4(0.44)	7(1.77)	<0.001[c]			0.077	-	-

Missing data	599(66.33)								
**Hospital Type Classification**									
Community health center	113(12.51)	65(16.41)							
District hospital	345(38.21)	146(36.87)	0.177	-	-	<u>0.006</u>	-	-	
General hospital of the city	445(49.28)	185(46.72)							
**Suspicious food (5 days before onset)**									
Yes	426(47.18)	121(30.56)	<u><0.001</u>	<u>2.030</u>	<u>1.580-2.611</u>	<u><0.001</u>	<u>2.006</u>	<u>1.447-2.781</u>	
No	477(52.82)	275(69.44)							
**Raising or contact with pets**									
Yes	210(23.26)	99(25.00)	0.497	0.909	0.691-1.196	-	-	-	
No	693(76.74)	297(75.00)							
**Travel history**									
Yes	15(1.66)	4(1.01)	0.368	1.655	0.546-5.025	-	-	-	
No	888(98.34)	392(98.99)							
**Restaurant dining**									
Yes	8(0.89)	4(1.01)	0.763	0.876	0.262-2.924	-	-	-	
No	895(99.11)	392(98.99)							
**Similar patients nearby**									
Yes	3(0.33)	2(0.51)	0.644	0.657	0.109-3.953	-	-	-	
No	900(99.67)	394(99.49)							
**Suspicious water**									
Yes	0(0.00)	1(0.25)	0.305	-	-	-	-	-	
No	903(100.00)	395(99.75)							
**Antibiotics take-in**									
Yes	52(5.76)	29(7.32)	0.283	0.773	0.483-	0.967	-	-	

No	851(94.24)	367(92.68)			1.238			
**Enteric disease history(in 6 months before)**								
Yes	4(0.44)	1(0.25)	1.000	1.758	0.196-15.873	0.458	-	-
No	899(99.56)	395(99.75)						
**Fever**								
Yes	80(8.86)	44(11.11)	0.219	1.286	0.872-1.896	0.034	0.626	0.406-0.964
No	823(91.14)	352(88.89)						
37.5℃≤t≤39.0℃	78(97.50)	43(97.73)	1.000	0.907	0.080-10.293	-	-	-
t>39.0℃	2(2.50)	1(2.27)						
**Nausea**								
Yes	404(44.74)	156(39.39)	0.078	1.246	0.979-1.585	0.782	-	-
No	499(55.26)	240(60.61)						
**Dehydration**								
No	883(97.79)	386(97.47)	0.732	0.874	0.406-1.887	0.332	-	-
Mild	20(2.21)	10(2.53)						
Moderate	0(0.00)	0(0.00)						
Severe	0(0.00)	0(0.00)						
**Vomiting**								
Yes	303(33.55)	90(22.73)	<0.001	1.718	1.307-2.255	<0.001	1.860	1.373-2.520
No	600(66.45)	306(77.27)						
1~2days	268(88.45)	80(88.89)	1.000	0.670	0.077-5.814	-	-	-
≥3days	5(1.65)	1(1.11)						
Missing data	30(9.90)	9(10.00)						
<3 times/day	176(58.09)	63(70.00)	0.036	0.576	0.346-	-	-	-

	≥3 times/day	126(41.58)	26(28.89)			0.961			
	Missing data	1(0.33)	1(1.11)						
**Abdominal Pain**									
	Yes	417(46.18)	162(40.91)	0.079	1.239	0.976-	0.354	-	-
	No	486(53.82)	234(59.09)			1.575			
	Persistent	22(5.28)	4(2.47)	0.182	2.20	0.746-	-	-	-
	Paroxymal	395(94.72)	158(97.53)			6.486			
**Abdominal Distention**									
	Yes	140(15.50)	54(13.64)	0.385	1.161	0.828-	0.380	-	-
	No	763(84.50)	342(86.36)			1.631			
**Diarrhea**									
	1~2 days	741(82.06)	308(77.78)	0.113	1.428	0.936-	-	-	-
	≥3 days	64(7.09)	38(9.60)			2.179			
	Missing data	98(10.85)	37(9.34)						
	<3 times/day	70(7.75)	11(2.78)	0.001	2.890	1.513-	-	-	-
	≥3 times/day	819(90.7)	372(93.94)			5.522			
	Missing data	14(1.55)	13(3.28)						
**Stool Appearance**									
	Watery	700(77.52)	295(74.49)						
	Loose	172(19.05)	74(18.69)						
	Mucous	14(1.55)	12(3.03)	0.332	-	-	-	-	-
	Bloody	1(0.11)	1(0.25)						
	Other	2(0.22)	1(0.25)						
	Missing data	14(1.55)	13(3.28)						
**Tenesmus**									
	Yes	9(1.00)	9(2.27)	0.076	0.433	0.171-	0.153	-	-

	No	894(99.00)	387(97.73)			1.099			
**Hyperactive bowel sound**	Yes	220(24.36)	96(24.24)	0.963	1.007	0.764-1.326	0.502	-	-
	No	683(75.64)	300(75.76)						

Notes.

Calculation of OR: NoV(+) to NoV(-)/ the first row to the second row.

Meaningful results were underlined. The P values which were close to the level of test ($p<0.075$) were in italics.

Cutoff=0.305.

To analyze more comprehensive clinical features, the data of less frequent stools (<3 times/day) were also included.

- Means "not done in the analysis".

[a] Outcome by the Pearson χ^2 test or the Fisher's test.

[b] Outcome by a multivariate logistic regression model.

[c] Compared among 18 groups in the "occupation" category.

[d] Only three of 18 kinds of occupations were included and analyzed in a logistic model(as a binary variable); others were interpreted as "missing data".
