# Supplementary material for: Epidemiology of norovirus infections among diarrhea outpatients in a diarrhea surveillance system in Shanghai, China: a cross-sectional study
Source: BMC Infect Dis. 2015 Apr 15;15:183. doi: 10.1186/s12879-015-0922-z (PMC4438334; doi:10.1186/s12879-015-0922-z)
Supplement: Additional file 3: Table S3. — Epidemiology and clinical features by examining NoV(+) and bacteria(+) patients. [file 12879_2015_922_MOESM3_ESM.pdf]

Table 3. Epidemiology and clinical features by examining NoV(+) and bacteria(+) patients

	**NoV(+)** n=903	**Bacteria(+)** n=432	**P[a]**	**OR[a]**	**95%CI[a]**	**P[b]**	**OR[b]**	**95%CI[b]**
**Detection Rate (%)**	22.91	10.96						
**Season**								
Spring(Mar.~May)	200(22.15)	58(13.43)						
Summer (Jun.~Aug.)	91(10.08)	189(43.75)	<0.001	-	-	<0.001	-	-
Autumn (Sep.~Nov.)	321(35.55)	161(37.27)						
Winter (Dec.~Feb.)	291(32.23)	24(5.56)						
**Age**								
0~4y	50(5.54)	45(10.42)						
5~18y	31(3.43)	11(2.55)						
19~44y	428(47.4)	181(41.90)	0.009	-	-	0.037	-	-
45~59y	194(21.48)	106(24.54)						
60~y	200(22.15)	89(20.60)						
**Gender**								
Male	489(54.15)	222(51.39)	0.344	1.117	0.888-1.406	0.226	-	-
Female	414(45.85)	210(48.61)						
**Residency**								
Local	805(89.15)	350(81.02)	<0.001	1.924	1.399-2.646	0.003	-	-
Immigrant	98(10.85)	82(18.98)						
**Occupation[d]**								
Officials/clerks	253(28.02)	80(18.52)				0.437	-	-
Kindergarten/home-stay children	47(5.20)	46(10.65)	<0.001[c]	-	-	0.033	0.090	0.010-0.822
Farmers/migrant laborers	4(0.44)	10(2.31)				0.008	0.180	0.051

								-0.643
Missing data	599(66.33)	296(68.52)						
**Hospital Type Classification**								
Community health center	113(12.51)	59(13.66)						
District hospital	345(38.21)	193(44.68)	0.031	-	-	0.170	-	-
General hospital of the city	445(49.28)	180(41.67)						
**Suspicious food (5 days before onset)**								
Yes	426(47.18)	163(37.73)	0.001	1.474	1.166-1.862	<0.001	1.686	1.266-2.244
No	477(52.82)	269(62.27)						
**Raising or contact with pets**								
Yes	210(23.26)	120(27.78)	0.078	0.788	0.607-1.022			
No	693(76.74)	312(72.22)						
**Travel history**								
Yes	15(1.66)	10(2.31)	0.410	0.713	0.318-1.600	-	-	-
No	888(98.34)	422(97.69)						
**Restaurant dining**								
Yes	8(0.89)	9(2.08)	0.114	0.420	0.161-1.096	-	-	-
No	895(99.11)	423(97.92)						
**Similar patients nearby**								
Yes	3(0.33)	0(0.00)	0.555	-	-	-	-	-
No	900(99.67)	432(100.00)						
**Suspicious water**								
Yes	0(0.00)	0(0.00)	-	-	-	-	-	-
No	903(100.00)	432(100.00)						
**Antibiotics take-in**								

Yes	52(5.76)	27(6.25)	0.722	0.917	0.567-1.481	0.815	-	-
No	851(94.24)	405(93.75)						
**Enteric disease history(in 6 months before)**								
Yes	4(0.44)	1(0.23)	1.000	1.918	0.196-15.873	0.939	-	-
No	899(99.56)	431(99.77)						
**Fever**								
Yes	80(8.86)	81(18.75)	<0.001	0.421	0.302-0.588	<0.001	0.428	0.288-0.635
No	823(91.14)	351(81.25)						
37.5℃≤t≤39.0℃	78(97.50)	79(97.53)	1.000	0.987	0.139-7.353	-	-	-
t>39.0℃	2(2.50)	2(2.47)						
**Nausea**								
Yes	404(44.74)	152(35.19)	0.001	1.491	1.176-1.890	0.001	1.735	1.247-2.412
No	499(55.26)	280(64.81)						
**Dehydration**								
No	883(97.79)	420(97.22)	<0.001[e]	-	-	0.544	-	-
Mild	20(2.21)	11(2.55)						
Moderate	0(0.00)	0(0.00)						
Severe	0(0.00)	1(0.23)						
**Vomiting**								
Yes	303(33.55)	91(21.06)	<0.001	1.892	1.445-2.475	0.006	1.620	1.149-2.286
No	600(66.45)	341(78.94)						
1~2days	268(88.45)	78(85.71)	0.659	1.374	0.262-7.246			
≥3days	5(1.65)	2(2.20)				-	-	-
Missing data	30(9.90)	11(12.09)						
<3 times/day	176(58.09)	47(51.65)	0.309	1.278	0.797-	-	-	-

	≥3 times/day	126(41.58)	43(47.25)			2.050			
	Missing data	1(0.33)	1(1.10)						
**Abdominal Pain**									
	Yes	417(46.18)	259(59.95)	<0.001	0.573	0.454-	<0.001	0.405	0.299-
	No	486(53.82)	173(40.05)			0.723			0.549
	Persistent	22(5.28)	5(1.93)	0.041	2.829	1.058-	-	-	-
	Paroxysmal	395(94.72)	254(98.07)			7.567			
**Abdominal Distention**									
	Yes	140(15.50)	55(12.73)	0.180	1.258	0.899-	*0.053*	-	-
	No	763(84.50)	377(87.27)			1.757			
**Diarrhea**									
	1~2 days	741(82.06)	324(75.00)	<0.001	2.073	1.420-	-	-	-
	≥3 days	64(7.09)	58(13.43)			3.030			
	Missing data	98(10.85)	50(11.57)						
	<3 times/day	70(7.75)	25(5.79)	0.215	1.347	0.840-	-	-	-
	≥3 times/day	819(90.7)	394(91.20)			2.160			
	Missing data	14(1.55)	13(3.01)						
**Stool Appearance**									
	Watery	700(77.52)	279(64.58)						
	Loose	172(19.05)	100(23.15)						
	Mucous	14(1.55)	26(6.02)	<0.001	-	-	-	-	-
	Bloody	1(0.11)	10(2.31)						
	Other	2(0.22)	4(0.93)						
	Missing data	14(1.55)	13(3.01)						
**Tenesmus**									
	Yes	9(1.00)	10(2.31)	0.081	0.425	0.171-	0.935	-	-

	No	894(99.00)	422(97.69)			1.054			
**Hyperactive bowel sound**	Yes	220(24.36)	89(20.60)	0.145	1.241	0.940-1.639	0.576	-	-
	No	683(75.64)	343(79.40)						

Notes.

Calculation of OR: NoV(+) to NoV(-)/ the first row to the second row.

Meaningful results were underlined. The P values which were close to the level of test (p<0.075) were in italics.

Cutoff=0.324.

To analyze more comprehensive clinical features, the data of less frequent stools(<3 times/day) were also included.

- Means "not done in the analysis".

[a] Outcome by the Pearson χ^2 test or the Fisher's test.

[b] Outcome by a multivariate logistic regression model.

[c] Compared among 18 groups in the "occupation" category.

[d] Only three of 18 kinds of occupations were included and analyzed in a logistic model(as a binary variable); others were interpreted as "missing data".

[e] Mann-Whitney U test was used to compare the ordinal variable.
